# Review of Histopathological and Molecular Prognostic Features in Colorectal Cancer

**DOI:** 10.3390/cancers3022767

**Published:** 2011-06-23

**Authors:** Ola Marzouk, John Schofield

**Affiliations:** Department of Cellular Pathology, Maidstone Hospital, Hermitage Lane, Maidstone, Kent ME16 9QQ, UK; E-Mail: olamarzouk@hotmail.co.uk (O.M.)

**Keywords:** colorectal cancer, prognostic factors, predictive factors, TNM classification, lymph nodes, micrometastases, sentinel node sampling, resection margins, molecular biomarkers, histomorphological factors

## Abstract

Prediction of prognosis in colorectal cancer is vital for the choice of therapeutic options. Histopathological factors remain paramount in this respect. Factors such as tumor size, histological type and subtype, presence of signet ring morphology and the degree of differentiation as well as the presence of lymphovascular invasion and lymph node involvement are well known factors that influence outcome. Our understanding of these factors has improved in the past few years with factors such as tumor budding, lymphocytic infiltration being recognized as important. Likewise the prognostic significance of resection margins, particularly circumferential margins has been appreciated in the last two decades. A number of molecular and genetic markers such as KRAS, BRAF and microsatellite instability are also important and correlate with histological features in some patients. This review summarizes our current understanding of the main histopathological factors that affect prognosis of colorectal cancer.

## 1. Introduction

The literature is replete with studies of the various clinical, pathological and molecular factors that affect the prognosis of colorectal cancer. Some factors are prognostically independent, while many are interdependent. This review summarizes our current understanding of the main histopathological factors that affect prognosis in colorectal cancer.

## 2. Search Methods for Identification of Relevant Studies

A primary search of the Medline database, using PubMed software, and based on the following inclusion criteria was used to identify relevant publications:

- Medical Subject Headings: Colorectal cancer and Prognostic, Table 1 cites articles and reviews retrieved;
- Limits Activated: Humans, English, French, German, Italian, Japanese, Publication Date from 1 January 1990 to 31 December 2010.

Preliminary search suggested that using “prognostic” rather than prognosis or prognostic factors seem to yield more relevant studies. This was followed by a secondary search for all relevant publications, identified from the references section of the articles retrieved by the primary search. Several further searches using expanded Medical Subject Headings were conducted, including but not limited to: Micrometastases, micrometastatic, sentinel node, tumor budding, tumor budding, differentiation, lymphovascular, peritoneal carcinomatosis, lymph node, TNM, metastases, resection margin, circumferential resection margin, microsatellite instability, MSI, KRAS, BRAF, molecular marker, biomarker.

The Cochrane library (including the Cochrane Central Register of Controlled Trials) and EMBASE databases were also directly searched in a similar fashion.

The limits activated arbitrarily included the last 20 years to make it manageable, although referenced earlier articles were retrieved as well following the secondary searches. Major languages were searched, but full articles were only sought in English language.

Finally, a judgment was made to include material pertaining to current clinical practice, limiting the review of data, which are currently in experimental setting.

## 3. Overview of Prognostic Factors

Prognosis of colorectal cancer has been extensively investigated over several decades. It was not until the 1930s that the first useful prognostic system for colorectal cancer was devised by Cuthbert Dukes, at St Mark's Hospital in London [1]. He realized that prognosis of colorectal cancer predominantly relied on its stage at presentation and treatment. While the tumor stage remains the most important factor, it has long been recognized that colorectal cancer is not a homogeneous disease. Individual patients with same stage tumors may have different long term prognosis and response to therapy. In addition, some prognostic variables are likely to be more important than others. This led to extensive research of other possible prognostic factors over the last eight decades, in attempt to improve identification of patients likely to have a poorer clinical outcome and therefore more likely to benefit from more aggressive treatment strategies.

Prognostic factors in colorectal cancer may be classified generally into:

1. Clinical factors: These include the poorer prognosis associated with presence of metastatic disease, perforated or obstructed primary tumors and a high CEA (Carcinoembryonic Antigen) levels. High CEA levels have been correlated with increased risk for recurrence and poor survival when found to be elevated preoperatively or postoperatively [2-4]. Prognosis was also poorer in patients with decreased CEA clearance after tumor resection [5,6]. The prognostic significance of preoperative CEA was independent of Dukes stage [7,8]. Similarly, Harrison *et al.* [9] found that preoperative CEA elevation in node-negative patients identified a subgroup of patients with poorer prognosis. They suggested that this subset of patients might benefit from postoperative chemotherapy;
2. Pathological factors: These include the pathological tumor stage (including involvement of lymph nodes, breach of serosa, peritoneal carcinomatosis, distant spread), primary tumor characteristics (including depth of tumor penetration in the bowel wall, histological subtype, histological grade and differentiation, tumor budding and its invasive front, venous and lymphatic invasion, perineural invasion, lymphocytic infiltration] and status of surgical resection margins (free or involved);
3. Molecular markers: These have been extensively studied in the last two decades;
4. The impact of preoperative and postoperative therapies. Preoperative radiochemotherapy can downstage tumors, facilitate resection and possibly influence long-term prognosis. Tumors showing complete response often have better prognosis. Likewise, postoperative adjuvant chemotherapy influence prognosis.

## 4. Tumor Stage

Tumor stage remains by far the most important predictor of prognosis and best guide to therapeutic decisions in colorectal cancer. The original Dukes staging classification was essentially an anatomic classification, based on the extent of tumor infiltration through the bowel wall, and presence or absence of lymph node involvement [1]. It correlated well with prognosis in these patients, but over the years it was modified and then supplanted by other classifications. This included his modified Dukes classification in 1944, introducing C1 and C2 subclassification, depending whether the apical node was involved [10], the addition of stage D by other authors to signify the presence of incurable disease including metastases to liver, lungs, bones, peritoneal seedings and omental implants, as well as locally irremovable tumors because of extensive local disease [11,12], the Astler-Coller classification [13], which classified tumors invading the muscularis into B1 and B2 (incomplete and complete penetration of muscularis) and confusingly classified tumors with lymph node metastases into C1 and C2, depending on the depth of tumor penetration of the muscularis (and not status of apical node as in the modified Dukes classification). Finally the American Joint Committee (AJC) and the Union Internationale Contre Le Cancer (UICC) joined forces to produce the TNM system [14], which attempt to record clinical and pathological data, guide therapy and forecast prognosis, all in one.

**Figure 1.**
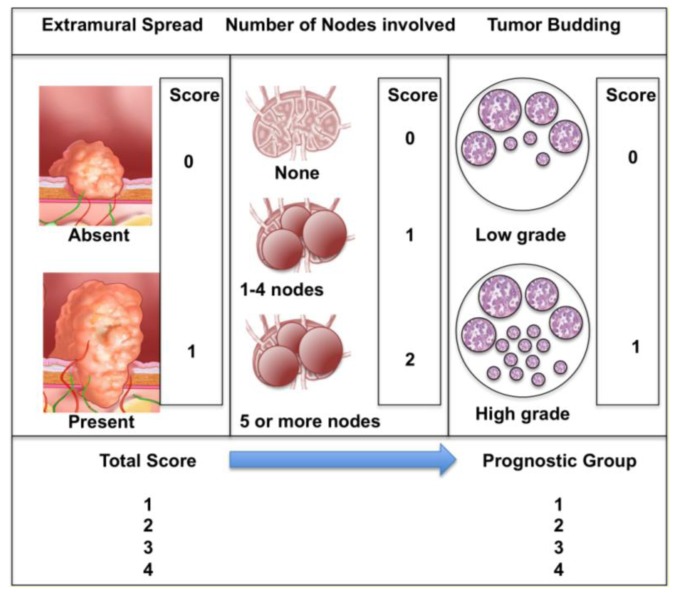
The Jass Classification (Ueno *et al.*, 2004 [17]): not in order Tumor is scored in three areas; extramural spread, positive nodes and tumor budding. The total score place it in a prognostic group.

Jass and colleagues [15,16] attempted to devise and validate a better prognostic system, adding the invasive front of the tumor as a prognostic biologic factor. This was subsequently modified [17] and simplified (Figure 1). They proposed using three variables only; presence of extramural spread, presence of positive nodes and extent of histologic tumor budding. They believed that their classification would forecast prognosis more accurately and would be a better guide to adjuvant therapy. Despite being validated [17], the system was a radical departure from the Dukes/TNM line, suffered from weak interobserver reproducibility and consequently has not been widely adopted [18].

Jass and Morson [16] criticized the TNM classification for sub-classifying the T stage based upon the extent of spread within the layers of the bowel wall (which appeared in their data to have little prognostic importance) as opposed to being applied to the extent of spread beyond the bowel wall, which they found to be of considerable clinical importance, particularly in the prediction of local recurrence. Recent data [19] have shown that there are prognostic differences even between early T stages. In addition, the Jass classification, by lumping together early T stages (T1, T2 and T3) failed to take into account the importance of such division for the indications for local excision, polypectomy and certain sphincter preserving options such as transabdominal transanal resections (TATA).

The TNM classification was conceived at the Institute Gustave-Roussy, Paris in the early 1950s [20], but it was not until 1987 that the UICC (Union Internationale Contre Cancer) and the AJCC (American Joint Committee for Cancer) unified their TNM classifications, which is currently into its 7th edition. The repeat revisions of TNM attest to both the dedication to include new knowledge and also the imperfect nature of staging classifications. The newer editions have generated a debate among pathologists regarding the validity of some of the changes and its potential impact on indications for adjuvant therapy [18,21,22]. Puppa and associates [18] suggested that staging classification needed to incorporate non anatomic tumor factors such as tumor budding, extranodal tumor deposits, molecular markers and treatment related factors. While all of these factors are likely to stratify the patients into more homogenous prognostic groups, it may make such systems too complex for wide adoption. Currently, therapeutic decisions seem to be well served by combining the use of TNM in addition to other known prognostic factors, although that makes accurate prognosis much more difficult. A comparison of the three last editions of the TNM classification is included in Table 2.

## 5. Lymph Node Involvement

Number of nodes examined is one of the most important prognostic factors and one of the primary indications of adjuvant chemotherapy. The presence of positive nodes as well as the number of nodes involved influences prognosis [24]. Therefore, ideally all lymph nodes should be harvested from colorectal cancer resections and examined histologically. This needs a lot of histopathology resources, which, may be unavailable to most pathology departments. In the UK, the Royal College of pathologists recommends examining a minimum of 12 nodes. Some published studies recommend higher number [25].

The number of lymph nodes recovered from resection specimens is dependent on several factors, starting with the surgeon's technique, tumor location and length of bowel resected [26] but mainly depend on the diligence and skill of the pathologist as well the time spent in searching for small nodes especially in fatty mesentery. There is sizeable variation between different pathology laboratories with regard to the number of lymph nodes examined histologically, as found in a recent Dutch nationwide study [27]. The latter study on 30682 patients, found that with increasing number of evaluated nodes, the risk of death decreased, either the result of quality of surgical resection (yielding more nodes for the pathologist) or because tumors with fewer nodes may have an innate reduced local response to their tumor leading to inferior prognosis.

For grossly positive lymph nodes, it is recommended that a representative sample be examined histologically. All grossly negative or equivocal lymph nodes should be submitted in their entirety for histopathology examination. In addition, if fewer than 12 lymph nodes were found after careful gross examination, it is suggested that additional techniques that aid in the macroscopic identification of lymph node, such as fat clearing, be considered. Likewise, examination of both halves of each node and preparation of more than one tissue level per paraffin block of nodes improves detection of metastatic disease [25].

Since nearly 20% of node negative colorectal cancer relapse, other means of finding nodal involvement is needed, to identify tumor markers that can distinguish between surgically cured patients and patients at higher risk of disease recurrence who may be candidates for intensive follow-up or adjuvant therapies.

Molecular markers have been proposed as means of finding metastases in morphologically negative nodes. Immunohistochemical methods to stain CEA and CK20 can be used but are labor intensive. Alternatively markers such as guanylyl cyclase C (GCC) (a marker uniquely expressed in apical cells of the GIT and by colorectal cancer cells) may aid the detection of colorectal cancer metastases in lymph nodes classified as negative by routine histopathologic examination (HP-negative) [28]. Such patients may be considered as pN0(mol+). Patients with HP-negative/GCC-positive CRC are at a greater risk of relapsing than patients classified with pN0 (mol−) disease. Haince and associates [28] found that GCC mRNA was detected in 8.0% of the 560 nodes (among 123 node negative patients) initially identified as HP-negative, whereas two repeat histopathologic examinations detected only 3.0% of these cases.

Desolneux *et al.* [29] published a study of 362 node negative patients followed for 140 months and found that multivariate analysis identified several independent prognostic factors that increased these patients' chances of relapse, including venous invasion, lymphatic invasion, perineural invasion and T4stage. Node negative patients with these pathologic features may benefit from using molecular markers to identify occult nodal metastases or alternatively may be candidates for adjuvant therapy. Lymphovascular invasion and perineural invasion were also found to be predictive of the risk of positive lymph nodes in 224 patients with T1 and T2 colorectal cancers [30]. Similarly, lymphatic invasion and high-grade focal dedifferentiation at the submucosal invasive front were important predictors of lymph node metastasis in 155 patients with nonpedunculated submucosal invasive colorectal cancers [31].

The risk of lymph node metastases increases with depth of tumor invasion. Haggitt classified tumor invasion of pedunculated polyps into four levels, with Level 4 invasion of the submucosa noted for its risk of lymph node metastases [32]. Suzuki and colleagues [33] found in addition that the width of submucosal invasion was significantly greater in node positive than in node negative patients, using 5 mm wide submucosal invasion to separate the groups in 65 Haggitt's Level 4 invasion patients who underwent radical resections between 1975 and 2000.

Tateishi and colleagues [34] found that lymph node metastases occurred in 16% of 322 patients with submucosal invasive T1 colorectal cancers. Multivariate analysis showed that lymphatic invasion, tumor differentiation, and tumor budding were significantly associated with lymph node metastasis. A smaller study on 48 patients [35] found tumor budding was the only independent factor associated with lymph node metastasis in cases of non-sessile submucosal invasive colorectal cancer. Analysis of 353 patients with T1 cancers treated at the Mayo clinic between 1979 and 1995, found 13% incidence of lymph node metastases. These were significantly more common in tumors with lymphovascular invasion, sm3 depth of invasion, and location in the lower third of the rectum [36].

### 5.1. Sentinel Lymph Node Sampling in Colorectal Cancer

Sentinel lymph node concept stipulates that lymph drainage from a primary tumor flows to a specific lymph node in the regional lymph nodes in orderly and sequential way. Biopsy of this node, once identified would accurately reflect whether the tumor has spread to lymph nodes or not. The concept does not take into account the percentage of patients with skip nodal metastases, but it has been utilized for breast cancers and melanomas for years. Skip metastases were found in only 3.6% of colonic and. 2.8% of rectal cancers in a series of 407 consecutive patients who underwent sentinel lymph node mapping (336 colonic, 71 rectal) [37].

The technique is certainly feasible, as shown in almost all published studies, and it is much easier in colonic cancer than in melanoma or breast cancer because the tumor, the lymphatics, and the sentinel nodes are all directly visible. It is clear, however, that both the experience of surgeons and pathologists improves the results [38,39]. In fact lack of experience has been blamed for the disappointing sentinel node yield in some results [40].

Sentinel nodes are identified by injected dyes, (Isosulfan blue, Patent blue, Vital blue, Indocyanine green) or radioactive tracer around the tumor. Dyes may be injected subserosally (around the tumor) intra-operatively in colonic patients. When attempted in rectal cancers the dye is normally injected endoscopically just before the operation into the submucosal plane (around the tumor). The technique incidentally may identify sentinel nodes outside the conventional regional node basin [40,41].

Sentinel nodes are harvested *in vivo* or *ex vivo* in colorectal cancer. The *in vivo* harvesting may be done during a staging laparoscopy for early colonic tumors (T1 and T2), especially if an endoscopic resective procedure or a limited segmental resection is thought to be appropriate [39]. If a radical resection is indicated, then the harvesting must be done *ex vivo*, by the surgeon himself or a ready waiting pathologist. The operative specimen is inspected to identify each blue-stained node, which are carefully dissected and sent separately to pathology.

The indications of sentinel node biopsy in colorectal cancer is mainly to stage (or upstage) the colorectal cancer and not to direct the lymphadenectomy (unlike melanomas and breast cancer, except in selected early T1 and T2 tumors. In these patients, with truly early tumors, it could theoretically have a role in helping select them for endoscopic resection. There is a risk of missing positive nodes (false negative) which at 10%–20% of many studies mandates that such a role must be investigated in a specific study of the technique in early stage tumors before recommending this approach [39]. In such cases, ideally sentinel node analysis should be performed intraoperatively to allow direct progress to the definitive radical resection, if it is positive.

Most studies claim that by focusing on two or three sentinel nodes (using multiple sectioning (multilevel sectioning) and immunohistochemistry staining, the pathologist may upstage some patients, altering the indications for adjuvant therapy [41-44]. Proponents of the technique suggest that it may upstage up to 15% of patients [40,45,46], although another multicentre trial that enrolled 91 patients, using multilevel sectioning of the nodes and examination by a single study pathologist, failed to predict nodal status in 54% of cases [47]. Since the natural history and the biological importance of nodal micrometastases and isolated tumor cells in colorectal cancer is still debatable, nobody knows whether using more focused examination of sentinel nodes (including molecular testing) is better than searching for and examining more nodes, a caution echoed by Redston and colleagues [48]. In addition, it is hard to predict the impact of adopting the technique on pathologist's workload.

A recent systematic review of the literature [39] of 52 studies on 3390 patients, found there is still insufficient evidence to support routine sentinel node sampling without further study. Most studies reported that sentinel node sampling is feasible in most colonic cancers, while rectal cancers are more problematic because the mesorectum is retroperitoneal and is usually bulky. In addition, *in vivo* retrieval of sentinel nodes (in early cases, which may be candidates for endoscopic or local resections) will transgress the mesorectal plane, which is vital for both good cancer resection of the rectum as well as the subsequent pathologic assessment of the circumferential margins. Furthermore, sentinel node sampling has higher false negative rates in rectal cancer [46] and usually fails in patients who had preoperative neoadjuvant radiochemotherapy [49]. Sentinel node sampling is also difficult in patients who had previous laparotomy and those with high BMI.

Some studies claimed it has no benefit as a reliable predictor of N0 status due to its relatively high false negative rate [50-52], emphasizing the need for further study [53]. Sentinel lymph node biopsy use in colorectal cancer is still limited, but promising [39,54,55]. There is a need for standardization of the technique, clarification of indications and enrolling patients in a large multicentre trial to define its role in colorectal cancer.

### 5.2. Lateral/Extended Lymphadenectomy

Rectal cancers located below the peritoneal reflection can spread laterally, beyond the mesorectum, through the lateral ligaments to the lateral pelvic lymph nodes located around the internal iliac vessels and the obturator hiatus [56]. Lateral lymph node involvement is thought to occur in 11%–18% of all patients [57-61], at least in patients in East Asia. The risk increases with deeply invasive tumors, tumors larger than 5 cm and those that are poorly differentiated [59]. Positive lateral nodes identify a subgroup with particularly poor prognosis with a 5-year survival reduced by about 25% [58].

Lateral pelvic nodes lie outside the boundaries of total mesorectal excision. This has led Japanese surgeons to perform additional lateral pelvic lymphadenectomy in patients with advanced low rectal cancer, a trend followed by Chinese and Korean surgeons [59,62]. This is rarely performed in the Western countries, at least partly because pelvic loco-regional recurrence following proper total mesorectal excision is far below the 11%–18% Figure [63]. Japanese surgeons have reported 5-year survival of 25%–50% after lateral pelvic lymphadenectomy in low rectal cancer [57,64,65].

#### Micrometastases

Twenty to 30% of node negative colorectal cancer patients develop recurrences and die of their disease. This is the result of the presence of micrometastases, which were not detected by current staging techniques. The presence of micrometastases has been detected in lymph nodes, bone marrow, liver and other organs. The exact biological significance of such micrometastatic disease and the need for systemic adjuvant therapies in these patients remain debatable, mainly because of the small sample size in most of the published series.

### 5.3. Nodal Micrometastases

Nodal micrometastases are defined as small deposits of tumor cells, less than 2 mm in diameter, in the regional lymph nodes. Micrometastases are only seen in a small minority of nodes using routine H and E stains. The sensitivity of detecting micrometastases is increased by histological serial sectioning, immune-histochemical techniques using monoclonal antibodies targeting different proteins such as CEA, cytokeratin 20 (CK 20), and cytokeratins AE1/AE3 (to detect the presence of epithelial cells in lymph nodes, indicating their metastatic nature), or employing polymerase chain reaction (PCR).

The significance of micrometastases is debatable and most studies suggest they may not affect survival as they are thought to be biologically distinct from macrometastases because they lack their own blood supply. Instead they get oxygen and nutrients by passive diffusion, which may limit their growth. They may remain dormant for long periods until the immune system eliminates them or until angiogenesis allows formation of new blood vessels that permit their growth [66].

Detection of micrometastases by means of molecular markers may play an important role in the future. A metanalysis conducted on 10 published studies with survival data, including 739 patients, found that RT-PCR upstaged 37% of patients and was associated with an absolute survival difference of 18.7% at 3 years. Immunohistochemistry upstaged 32% of the patients and again showed tendency to worsened survival, although it did not reach statistical significance [67].

#### Peritoneal carcinomatosis

Peritoneal carcinomatosis (PC) is seen in about 10%–13% of patients with colorectal cancer during the initial operation [68,69]. It is seen more frequently in colon cancer than among patients with rectal cancer, because of the higher likelihood of the cancer cells being shed into the peritoneal cavity following the serosal penetration. These patients occasionally present with ascites and weight loss before the discovery of the colorectal primary.

The prognosis of these patients is dismal. In patients with stage IV, the presence of peritoneal carcinomatosis is associated with a significant reduction in survival, from 18.1 months to 6.7 months [70]. Treatment has traditionally been palliative with systemic chemotherapy when diagnosis is established before surgery. Most patients are discovered intraoperatively and surgeons may elect to do standard operations, segmental resections or defer surgery altogether unless the patient faces imminent obstruction, referring patients to chemotherapy and palliative care.

Peritoneal carcinomatosis is often associated with metastatic disease, although the peritoneal cavity appears to be the only site of the disease in about 25% of patients. Several groups have advocated the use of cytoreductive surgery and hyperthermic intraperitoneal chemotherapy (HIPEC) as a means of improving survival in these patients. This treatment, however, is associated with significant morbidity and mortality [71] A randomized trial from the Netherlands that compared cytoreduction surgery plus HIPEC with systemic chemotherapy plus palliative surgery found that patients in the former group exhibited a statistically significant improvement in median survival (22.3 months *versus* 12.6 months [72] Cytoreductive surgery plus HIPEC seems to be a viable option for the treatment of peritoneal carcinomatosis. Patient selection for these aggressive procedures remains a major issue, given the substantial morbidity and mortality associated with them.

Peritoneal carcinomatosis from colorectal cancer was traditionally associated with a 6-month survival. This dire survival outcome reflects the failure of conventional systemic chemotherapy and palliative surgery to control disease. A surgical approach combining cytoreductive surgery (CRS) and hyperthermic intraperitoneal chemotherapy (HIPEC) is beginning to gain increasing acceptance in the oncological community as a treatment option for patients with colorectal cancer peritoneal carcinomatosis. Prognosis in these patients depends on the extent of carcinomatosis, the ability to achieve a complete cytoreduction and the tumor biology.

## 6. Primary Tumor Characteristics

### 6.1. Early Colorectal Cancer: Malignant Polyps and EARLY Flat Cancers

The advent of colonoscopy and colonoscopic resection of colorectal lesions in 1969 [73] was followed by increasing removal of polyps with invasive cancers in the following and subsequent decades. This ushered a new era for pathologists, gastroenterologists and surgeons, trying to define the indications and prognosis of these endoscopically removed lesions. Recently, improved endoscopic techniques, national screening programs and the categorization of small colonic lesions enhanced the pace of studying these early lesions.

Invasive adenocarcinoma, by definition means that cancer cells have invaded the muscularis mucosae and the submucosa [74]. Early colorectal cancers are defined as superficial invasion of the submucosa, all being early TNM T1 stage [75]. This term was coined to encompass malignant polyps and nonpolypoid early cancers that may be candidates for endoscopic resections.

Although advances in colonoscopic visualization technology, pit morphology and endoscopic ultrasound assessment are important, it is the pathologic report that remains central to decision whether the patient will be offered a radical cancer resection or merely endoscopic follow-up. This depends entirely on the risk of local recurrence, lymph node and distant metastases. Pathological assessment may be hampered by the not infrequent piece-meal resection of polyps, which may make the evaluation of the depth of invasion and specimen orientation challenging. Properly marked and orientated specimens are thus essential [76].

The depth of invasion and local resection margins of these early cancers are crucial in determining the risk of local recurrence. It also underlines all classifications of these early lesions. Haggitt described his classification for pedunculated polyps in 1985 [32], followed by the Kikuchi classification [77] for sessile lesions in 1995. The Haggitt's classification classified early colorectal cancer into pedunculated and sessile lesions. Haggitt classified polyps depending on the invasion into four levels (invasion of head, neck, stalk and beyond stalk). Levels 1, 2 and 3 carries a low risk of lymph node metastases and can be treated endoscopically. Level 4 invasion and rectal location (had more Level 4 lesions) were the only statistically significant adverse prognostic factors in the 64 patients studied with early invasive cancers in polyps. It is suggested that Level 4 patients need radical resections. Initially, Haggitt considered all sessile lesions with submucosal invasion to be equivalent to Level 4. However subsequent studies found this to be inaccurate and Kikuchi and associates came up with their classification for sessile polyps, quantifying both vertical and horizontal submucosal invasion. Kikuchi classification subdivide the submucosa into three thirds; superior third (Sm1), medium third (Sm2) and lower third (Sm3). Sm1 is further subdivided into three subtypes (Sm1a, Sm1b, Sm1c) depending on horizontal spread in relation with the tumor size. In a group of 182 patients (64 Sm1, 82 Sm2 and 36 sm3), all local recurrences (4 patients) and lymph node metastases (13 patients) occurred in Sm2 and Sm3 disease, suggesting that only Sm1 patients can be treated endoscopically. A recent review of the literature found the risk of lymph node metastases was 1%–3% in Sm1, 8% in Sm2 cancers and 23% in sSm3 cancers [78]. These recommendations were subsequently refined by other pathologists who emphasized that tumors with any additional adverse characteristic (unfavorable histology), such as poor differentiation, lymphatic or venous invasion and a polypectomy resection margin <2 mm, should be offered resection even in Haggit Level 1–3 and Kikuchi Sm1 [79,80].

The Japanese classified the gross morphology of nonpolypoid early cancer as slightly elevated, flat, depressed and laterally spreading [81]. Most need dye spray techniques and pit pattern analysis to detect them during colonoscopy. The depressed variety is much more likely to show deeper submucosal invasion. It became apparent there was a huge gulf between Japanese classifications and classifications used by western pathologists, which led to consensus building workshops, including the Vienna Classification [82] and the subsequent Paris classification [83].

Analysis of polypectomy resection margin showed that in pedunculated polyps, the risk of local recurrence depends on presence of uninvolved stalk and will be 0%–2% when the margin is >1 mm. By contrast with an involved resection margin, or when less than 1 mm, the risk of recurrence is 21%–33% [84]. Ueno and associates [85] studied 292 early invasive cancers and found that unfavorable tumor grade, vascular invasion, and tumor budding were high risk factors for nodal metastases with 0.7%, 20.7%, and 36.4% in the no-risk, single-risk, and multiple-risks groups respectively. They also suggested that an absolute thickness of submucosal invasion >2 mm in depth or 4mm in width were significant risk for recurrence. Currently the Royal college of pathologists suggests taking into consideration; Haggitt's, Kikuchi classifications, presence of lymphovascular invasion, poor differentiation and the depth and width of invasion, in order to offer advice whether endoscopic resection is appropriate or not.

### 6.2. Tumor Depth of Invasion (T stage)

The tumor depth of invasion is important. Submucosal invasion allows vascular and lymphatic infiltration with subsequent nodal and distant metastases. As mentioned previously, the absolute thickness of submucosal invasion predicts local recurrence and lymph node metastases after endoscopic resection [85]. Likewise it is one of the most important prognostic factors in larger tumors [15-17]. This is discussed under staging.

### 6.3. Tumor Size

Although some authors believe that tumor size have no effect on prognosis [86-88], others believe that tumor size partly influences prognosis, although not as much as other factors. This point was illustrated by a Danish study on 468 radically operated patients (260 Dukes' B and 208 Dukes' C) [89], whereby multivariate analysis suggested the prognostic significance of the size of the primary tumor seems to be different between Dukes' B and C. Large tumors presenting without lymph node metastasis may have less aggressive natural history, while in Dukes' C tumors, the influence of tumor size is consistent with what is seen in most other cancers: Increasing tumor size is associated with decreased loco-regional control.

It is also known that polyp size increase the risk of its malignant potential [75]. Bigger tumors are more likely to be more deeply invasive and may invade neighboring organs. Rectal tumor size may also influence the risk of lateral pelvic nodal metastases in low rectal cancer [59].

### 6.4. Histological Types and Variants

Most colorectal cancers are adenocarcinomas of which mucinous and signet ring adenocarcinoma constitute approximately 10%, with signet ring carcinoma comprising 1%–2.4% [90]. Mucinous cancers are defined histologically by the presence of abundant extracellular mucin, with more than 50% of the tumor mass being mucinous. Signet ring cancers have intracellular mucin pushing the nucleus to one side [87].

The impact of mucinous histology on prognosis is controversial. While it is associated with more advanced stage, metastases and recurrence, these differences are dependent on tumor stage. A mucinous morphology has not been found to be an independent prognostic factor on multivariate analysis [91]. On the other hand, the presence of signet ring morphology has been found to be an independent poor prognostic factor on multivariate analysis in a large study including 2,764 cases of sporadic colorectal cancers [92]. Signet ring carcinoma was also associated with more advanced stage at diagnosis, higher incidence of lymphovascular invasion, lymph node and liver metastases as well as higher rate of recurrence. It has been reported that it has different molecular pathways, which may explain its aggressiveness [93].

Mucinous cancers are associated with microsatellite instability (MSI) both sporadic and hereditary nonpolyposis syndrome as well as colorectal cancers with high degrees of methylation (CIMP).

Despite the fact that many signet ring carcinomas are associated with microsatellite instability-high and features common to other microsatellite instability-high cancers: older age group, female preponderance, right-sided location, Crohn's-like reaction and numerous tumor-infiltrating lymphocytes, its microsatellite instability status does not appear to be a significant predictor of survival [90].

Medullary carcinoma and serrated adenocarcinoma are two variants of colorectal cancer, which are also associated with particular molecular pathways. Medullary carcinoma is invariably associated with MSI while serrated adenocarcinoma is characterized by excess of methylation.

Medullary carcinomas are usually bulky, located in the proximal colon and have female predominance. It shows an expansile, well circumscribed growth pattern and is formed of solid sheets of undifferentiated cells with round nuclei, prominent nucleoli and is associated with a Crohn's like reaction. They show reduced expression of CK20 [94] and lack of expression of CDX2 [95]. Medullary carcinomas are often diploid, MSI high whether sporadic or familial and have a favorable prognosis with reduced incidence of lymph node involvement [96,97].

Serrated adenocarcinoma is a recently recognized entity with distinct morphological features accounting for approximately 7.5% of all colorectal cancers and 17.5% of proximal tumors [98]. Their precursor lesions are traditional serrated adenomas, hyperplastic aberrant crypt foci, sessile serrated adenomas and mixed polyps, a pathway characterized by strong association and early involvement with BRAF mutations and a CIMP-high phenotype [98-100] and subsequently low or high microsatellite instability. The majority being MSI low with traditional serrated adenoma as their precursor lesion and a minority are MSI high with sessile serrated adenoma and mixed polyps as their precursor lesions [74].

Morphological features of these tumors have been recognized. Serrated adenocarcinomas show a constellation of features some of which superficially resemble hyperplastic polyps; however glandular serration in isolation is non-specific. These features include serrated epithelium with eosinophilic cytoplasm, vesicular nuclei with prominent nucleoli and retained polarity or prominent mucin production with serrated pattern and abundant eosinophilic cytoplasm forming floating balls or papillae as well as a less well differentiated appearance showing a more trabecular growth pattern, but with similar cytological details [101]. The implication of the serrated pathway in the development of colorectal cancer is of particular importance as its precursor lesions could be easily missed in the context of bowel screening.

Undifferentiated colorectal cancers are uncommon variant accounting for less than 1% of all tumors. They show no features of differentiation, lack mucin production and lack the features characteristic of medullary carcinoma and are associated with poor prognosis [102].

### 6.5. Tumor Degree of Differentiation

Colorectal cancers degree of differentiation has long been reported, but is rarely used as an independent prognostic factor. This has been largely the result of the inherent variability in reporting it, as a result of using different grading systems and the subjective nature of such assessment. The college of American pathologists consensus statement in 1999 [86] recommended using two grades only (high and low grade), with gland formation being the only feature used to assign the grade (<50% gland formation defines high grade) to reduce inter-observer variation and retain or improve prognostic significance. They also suggested excluding medullary and mucinous cancers from assigning a differentiation grade.

The current predominantly used system divides adenocarcinoma into three grades of differentiation based on the degree of tubular formation. Grade I (well differentiated), is formed mainly of simple tubules with relatively uniform nuclei with no pronounced nuclear stratification or loss of polarity. Grade II (moderately differentiated), shows simple or complex irregular glands with barely maintained, disorganized or lost nuclear polarity. To define Grade III (poorly differentiated) there should be either predominant loss of tubular differentiation or irregularly folded small tubules with loss of nuclear polarity. To assign a tumor Grade III, the poorly differentiated element should constitute more than 50% of the tumor rather than a minor component, excluding the commonly seen disorganized glands at the advancing edge of the tumor. Multivariate analysis of prognostic factors show histologic grade to be independently predictive of survival, high grade tumors (poorly differentiated and undifferentiated) being associated with increasing wall penetration more nodal and distant metastasis, and worse prognosis [87,103,104]. Recently, it was recommended (that grading should be based on the worst area even if it was not predominant [102], signet-ring cell carcinoma be assigned a Grade III while poorly differentiated and undifferentiated carcinoma a Grade IV. Undifferentiated carcinoma should be distinguished from the relatively undifferentiated medullary carcinoma that is not associated with adverse prognosis and recommended that medullary carcinoma not be graded [74].

### 6.6. Presence of Lymphovascular Invasion

Tumor invasion of lymphatics and blood vessels has been long recognized as independent prognostic factor on multivariate analysis, increasing the risk of lymph node and distant metastases [89,105]. It is an important indication of adjuvant chemotherapy, independent of stage. A study of 703 colorectal cancer patients showed that the 5-year survival rate was significantly worse and liver metastases developed more frequently when venous invasion was present. Invasion of extramural veins was found to be prognostically more significant than intramural venous invasion. Invasion of large (thick-walled) veins was also prognostically more significant than invasion of small (thin-walled) veins [106]. Only minimal venous invasion is required for the seeding of distant metastases [107], which emphasizes the importance for looking carefully for lymphovascular invasion.

Extramural vascular invasion is reported in 10%–90% in the literature [107], depending on the stains used and whether it is employed in all blocks or selectively. The use of an elastic stain on all tumor blocks results in the highest pick-up of venous invasion. It is recommended that at 3–5 blocks of tumor should be examined for lymphovascular invasion. Identification of tumor cells within an endothelial-lined spaces or surrounded by an elastic lamina is diagnostic of vessel invasion [18]. In addition, morphological clues on H and E-stained slides of venous invasion has been described including close association with a vein, the “protruding tongue”: When there are tongues of tumor protruding beyond the lower edge of the advancing tumor into the pericolic fat and the “unaccompanied artery” signs: When there is a relatively well circumscribed tumor nodule adjacent to an artery, and no vein seen [108].

The incidence of lymphovascular invasion increases with tumor stage and poor differentiation. Extramural vascular invasion is associated with a significantly worse prognosis and has been recognized as an independent prognostic marker in some studies with multivariate analysis [89,105,106].

### 6.7. Presence of Perineural Invasion

Perineural invasion is found in up to 33% of colorectal cancers at the time of resection [109], but may be grossly underreported in many series. On re-review of 269 consecutive resection specimens, perineural invasion was found in 22% (opposed to less than 0.5% in the initial pathology reports) [110].

The presence of perineural invasion is associated with higher rate of metastatic disease, recurrence and reduced survival. It has been increasingly recognized as an important independent prognostic factor in colorectal cancer on multivariate analysis in several studies. It was found to be the strongest prognosticator on multivariate analysis in a Danish study of 468 patients [89].

Metastases were found in 72.7% of patients with perineural invasion, as opposed to 27% of those without perineural invasion [111] on retrospective analysis of 77 patients with colorectal cancer. In a study of 563 curative anterior resections, the presence of perineural invasion was associated with a 27% cancer-specific 5-year survival rate *versus* a 78% 5-year survival rate in perineural invasion negative cancers [105]. Another study reported 16% cancer-specific 5-year survival rate *versus* a 65% 5-year survival rate in perineural invasion negative cancers [110]. In the same study node-negative disease with patients without perineural invasion had 5-year disease-free survival rate of 56% *versus* 29% for patients with node-negative, perineural invasion-positive tumors. Similar results were seen in other studies looking at prognostic significance of perineural invasion in lymph node-negative disease [112]. Some authors therefore suggest that perineural invasion should be another indication of adjuvant chemotherapy [109,113].

### 6.8. Tumor Budding and Invasive Front

The concept of tumor “budding” was re-introduced to colorectal pathology by Morodomi and colleagues in 1989 [114] and Hase and associates in 1993 [115], although first described in 1954 by Imai [116] as tumor sprouting. Tumor budding is generally defined as *An isolated single cancer cell or a cluster composed of fewer than five cancer cells, which were observed in the stroma of the actively invasive region*. It can be detected at high magnification by conventional hematoxylin-eosin (H and E) stain with high reproducibility. This is usually based on the number of such cells within a microscopic field of X250.

The dissociation of cancer cells from the main tumor body may be the first event in invading local and systemic tissues. Tumor budding is thought to be the morphologic expression of this [115]. Tumor buds show pseudopodia like cytoplasmic protrusions, identified by immunohistochemistry with pan-cytokeratins. These may be associated with tumor cell motility and increased invasion ability and underline the tumor capacity for metastasis [117,118]. Tumor budding is thought to be a feature specific for tumors showing an infiltrating growth pattern [119] and thus represent an important sign of tumor aggressiveness.

Tumor budding has not yet been accepted as a routinely examined pathologic parameter for colorectal cancer, yet it has been shown to be an independent prognostic factor in several studies, but not all.

Hase and his colleagues [115] looked at the prognostic significance of tumor budding in 663 patients who underwent curative resection for colorectal cancer between 1970 and 1985. They classified tumor budding to BD-1 (None or mild), which occurred in 74.4% of their patients and BD-2 (moderate or severe), which occurred in 25.6% of patients. Recurrence occurred in 20% and 71.1% of these two groups respectively (P < 0.005). The 5-year survival rate was worse in BD-2 than in BD-1 (22.2% *vs.* 70.7% (P < 0.001). Similarly the 10-year survival rate was also worse in BD-2 than in BD-1 (13.8% *vs.* 50.6% (P < 0.001). Even more significantly, the five-year survival rate of Dukes B patients with BD-2 lesions was worse than that of Dukes C patients with BD-1 cancers (29.1% *vs.* 66.2% (P < 0.001). Additionally, there was no difference in five-year survival among BD-1 patients with either Dukes B or C lesions (68.3% *vs.* 66.2%). They suggested that patients with more severe budding not only needed closer follow-up, but also possibly should receive adjuvant chemotherapy, regardless of their Dukes stage.

Ueno and colleagues [17] also found that high grade tumor budding was an independent prognostic factor (*together with lymph node involvement and presence of extramural tumor spread*) in multivariate analyses of two cohorts of 638 and 476 patients undergoing potentially curative resections for colorectal cancer. In both cohorts the 5-year survival rate was much lower (41%–43%) in patients with high grade budding, compared 83%–84% in patients with low-grade budding. They also proposed a new version of the Jass prognostic staging system, based on these three pathologic variables; extramural tumor spread, number of lymph nodes involved and tumor budding (Figure 1).

A significant association between tumor budding and lymph node positivity has been consistently demonstrated correlating with tumor aggressiveness and more advanced TNM stage.

Okuyama *et al.* [120], studied the significance of tumor budding and lymphovascular invasion in 101 pT1 or pT2 well-differentiated colorectal resection specimens, using sections stained with hematoxylin-eosin. They found that budding in combination with lymphovascular invasion was a predictive marker of lymph node metastasis in curatively resected pT1 or pT2 colorectal cancer and recommended that the presence or absence of budding should be looked for routinely in even pT1 or pT2 well-differentiated colorectal adenocarcinomas.

Sy *et al.* [121] studied 477 patients with long follow up after potentially curative resection of node positive colon cancer and although they found that the 5-year survival rate decreased from an overall of 51% in tumors with high budding (>9) to 33.9% in tumors with low budding, this was not independent of other prognostic factors such as venous invasion and involvement of serosa.

Zlobec and Lugli [122] hypothesised that tumor progression and prognosis in patients with colorectal cancer depended on the balance between pro-tumor factors such as budding and anti-tumor factors such as T lymphocytic infiltration at the tumor invasive front. Evidence seems to suggest that the presence of tumor budding or an infiltrating growth pattern is inversely correlated with the presence of immune and inflammatory responses at the invasive tumor front [16].

Using double immunostaining for pan-cytokeratin (CK22) and anti-CD8 antibody highlighting “attackers” (tumor buds, brown) and “defenders” (CD8+ T-lymphocytes, red) at the invasive front of a colorectal cancer with infiltrating tumor border configuration.

Tumor budding may also prove useful in prediction of response to anti-epidermal growth factor receptor based therapies (cetuximab or panitumumab) in metastatic colorectal cancer patients (along with K-RAS). A small preliminary study on 43 such patients found that all patients with high grade tumor budding were non-responders [123].

### 6.9. Lymphocytic Infiltration

Lymphocytic infiltration in colorectal cancers may be of prognostic significance. Several studies have suggested that it is associated with better prognosis. This possibly reflects an anti-tumor immune response, which is mediated by T cells. It may play a role in preventing tumor recurrence and metastasis. Lymphocyte infiltration appears to be more prominent in MSI-high colorectal cancer [124], possibly because these tumors are more immunogenic with their increased rate of mutations. Quantification and characterization of lymphocytic infiltration of tumors is variable on HE sections and should normally be done immunohistochemically.

Naito and associates [125], studied CD8+ T cells infiltration of colorectal cancer in 131 randomly selected cases, who had been followed up for a minimum of five years. The T cell infiltration was classified into three groups; (a) infiltration within cancer cell nests; (b) infiltration of the cancer stroma; and (c) infiltration along the invasive margin (tumor-host interface). CD8+ T cells were distributed mainly along the invasive margin and in the stroma, but it was the CD8+ T cells within cancer cell nests that were most significantly associated with a better survival of patients on multivariate analysis. The impact on survival was found to be similar to that of Dukes' staging. Many of these T cells were activated cytotoxic phenotype as evidenced by the detection of Granzyme B+ cytoplasmic granules in lymphocytes within cancer cell nests. As there was significant correlation between the degree of CD8+ T-cell infiltration within cancer cell nest and Dukes' staging, the authors stipulated that T cells may function not only locally but systemically to suppress micrometastasis after being activated in the cancer tissue.

By contrast Menon *et al.* [126] found that marked infiltration of CD8+ and CD57+ cells at the advancing tumor margin were independent prognostic factors for a longer disease-free survival on multivariate analysis of unselected 93 colorectal cancers. CD8+ and CD57+ cells infiltration was significantly higher in MSI-high tumors.

In a large study involving 371 consecutively sampled colorectal cancer patients, Chiba and associates [127], found that the number of intraepithelial CD8+ T cells, counted at the most densely distributed areas, was an independent prognostic factor in colorectal cancer. This appeared to influence prognosis mainly in the long term and they also suggested that it might reflect the presence of systemic immunosurveillance against micrometastasis of cancer cells.

Deschoolmeester and colleagues [128] investigated the possible role of a body cellular antitumor response mediated by cytotoxic lymphocytes. They analyzed the presence of CD3+, CD8+ infiltration and the expression of granzyme B and its prognostic significance in 215 colorectal cancer patients and found that intra-epithelial infiltration of CD3+ and CD8+ T lymphocytes and stromal infiltration of CD3+ lymphocytes had a major impact on the patients' overall survival in the univariate analysis, independent of their association with MSI-status. In addition, it was also demonstrated that there was an important disease specific survival advantage for patients with microsatellite stable (MSS) tumors containing intraepithelial CD8+ tumor infiltrating lymphocytes. When samples were analyzed for colon cancer and rectal cancer separately, the results of the overall population were confirmed in colon cancer only. When entered into a multiple Cox regression analysis adjusting for other possible important confounding factors, the strong impact of lymphocyte infiltration on overall survival was not maintained.

### 6.10. Tumor Angiogensis

Angiogenesis is thought to be associated with tumor aggressiveness and poorer prognosis. Angiogenesis provides nutrients for growth of tumors and increase the likelihood of tumor cells entering the blood stream, facilitating metastases. Angiogenesis has been studied in cancer patients by Immunohistochemical methods; using micro-vessel density (at the invasive margin of the tumor), identified by CD34 or CD31 staining and antibodies to vascular endothelial growth factor (VEGF) expression. High micro-vessel density and/or vascular endothelial growth factor expression was found to be predictor of poor survival in colorectal cancer in meta-analysis of published data [129]. Long course preoperative radiotherapy appears to significantly decrease the micro-vessel density in rectal cancer when compared with no treatment or short course radiotherapy [130].

## 7. Extramural Cancer Deposits

Extramural cancer deposits are discrete cancer nodules seen deposited in pericolonic and perirectal fat. Their presence imply that aggressive cancer cells have succeeded in spreading discontinuously, presumably through lymphatic or venous channels [131] and also entail poorer prognosis, irrespective of their classification [132]. They have generated a great deal of interest and controversy over the last 20 years. The TNM system classified them according to the 3 mm rule in TNM 5 and then according to contour in TNM 6, but there is yet no general consensus on how to classify this histologically heterogenous group, which makes interpretation of published results rather difficult.

Their prevalence has been reported from as low as 5% to as high as 65% in pericolonic and perirectal fat [132]. It has been argued recently [18,131,133] that the prognostic significance of these heterogeneous lesions may be attenuated by lumping them together and an effort should be made to classify them. One proposal is to use a combination of different pathologic features including shape; morphologic appearance, presence of lymphoid tissue not organized in residual lymph node structure and the degree of pre-existent anatomic structures, including lymphatic vessels, veins, and nerves into three types; tumor deposits confined to a vascular spaces, tumor deposits with no evident association with veins and nerves and tumor deposits in close association with extramural venous and perineural invasion. The latter have worse prognosis. Further work and agreement on these classifications is awaited.

## 8. Resection Margins

### 8.1. Distal and Circumferential Margins

Clear surgical margins are one of the most important prognostic factors in colorectal cancer. Reporting margins, not only predicts the risk of local recurrence, but also guide postoperative therapy. Understanding the way colorectal cancer spreads inside and outside bowel wall as well as the surgical techniques used is important for accurate reporting of margins.

The intramural spread of colorectal cancer along the wall has been studied for over 100 years. Quer and associates [134] as well as Grinnell [135] published their work in the mid 1950s that recommended aiming for 5 cm distal margin. This influenced the surgical world until this was challenged in 1983 by two well designed studies on the spread of rectal cancer [136,137] that recommended that distal margins do not need to exceed 2 cm, expect perhaps in poorly differentiated tumors. This has become the gold standard and played a significant role in the adoption of sphincter saving resections in mid rectal cancers. Even this is being challenged by recent publications which suggest that local control and survival do not seem to be compromised by 1 cm distal resection margin, after preoperative chemoradiotherapy, in otherwise favorable tumors [138-140]. This trend is likely to continue in view of the recent popularity of using Transabdominal Transanal (TATA) resections, whereby the rectum is transected distally (trans-anally), usually at the dentate line or just above it, dissection is started distally in the intersphincteric plane for 1–2 cm before transecting the bowel [138,141-147], although a recent study [148] on 672 patients treated between 1991 and 2003 suggested that close distal margins may increase recurrence.

Routine histological examination of doughnuts may not be necessary [149,150], as they very rarely show any pathology.

While distal margins in rectal cancer seemed less important, than in some other gastrointestinal cancers, circumferential margins proved to be extremely important for prediction of local recurrence and survival, especially in rectal cancers. Treatment of rectal cancer has undergone a significant change in the last three decades, in the adoption of newer surgical techniques, in pathological analysis, in adjuvant radiochemotherapy and in improving the training and the quality of surgery performed. Adoption of sphincter saving resections in the early 1980s was followed by the publication of the concept of total mesorectal excision [151], which was very widely accepted. Importantly, this also coincided with landmark pathologic publications [152,153] that proved that circumferential margins were important for recurrence and survival and thus helped in the worldwide adoption of resecting the rectum within its fascial envelope (total mesorectal excision) in combination with the increasing use of radiochemotherapy.

During the subsequent two decades, many publications confirmed the significant effect of positivity of the circumferential margins on recurrence and survival including a systemic review of more than 17,500 patients [154]. More recently, similar findings were reported in colonic cancer, especially the right side [155,156].

It is interesting that while progress was made in performing surgery for the upper and middle rectum, the lower rectal tumors that needed to be treated with abdominoperineal resections were faring badly [157-160]. This turned out to be a result of the enthusiasm of surgeons for sphincter saving resections, thereby compromising the cylindrical abdominoperineal resection, first laid by Sir Ernest Miles for a (APR) in 1908, thus performing APR resection with a “waist”, usually at the tumor site, as the dissection “coned” towards the pelvic floor. The MRC CR07 Trial addressed this by definitions of the quality of mesorectal excisions in anterior resections and APR [161]. Training in performing a cylinderical APR [162], especially in the prone position and using extralevator technique seem to have improved the results [162-164].

Other publications showed the poorer prognosis associated with invasion of adjacent organs [165-167].

### 8.2. Local Excisions Resection Margins

This has been discussed fully under Section 6.1.

## 9. Port Site and Extraction Site Recurrence

Initial reports of occurrence of port site and extraction site recurrence after laparoscopic resections have not been borne out by subsequent systematic reviews [168,169]. Nevertheless, these remain a potential risk when operating, particularly on T4 tumors. Recently the protective value of double plastic protection has been suggested as a routine maneuver in laparoscopic operations [170].

## 10. Molecular Markers

### 10.1. Molecular Genetics of Colorectal Cancer and Relevance to Prognosis

Significant progress in our understanding of the complex molecular genetics of colorectal cancer has been made in recent years, although a lot more awaits discovery, it is already affecting therapeutic decision making [171]. We now know that colorectal cancers have heterogeneous molecular profiles and some associated with recognizable tumor phenotypes. Malignant transformation is believed to involve mostly stepwise and sequential accumulation of a multitude of genetic mutations (directly involving the DNA), including the activation of proto-oncogenes and the inactivation of tumor suppressor genes as well as epigenetic mutations (DNA silencing by hypermethylation) [172]. As a result of this, malignant cells escape from normal homeostatic regulation of cellular proliferation and acquire a limitless ability for cell proliferation and survival. The mechanisms by which cancer cells acquire these capabilities vary considerably between tumors of different types; most involve alteration of signal transduction pathways (such as Ras-Raf-MEK-extracellular signal-regulated kinase 1 and 2 (ERK1/2) pathway). Recent studies indicate that the genomic landscape of colorectal cancer is far more complex and heterogeneous than previously thought [173,174]. In this review we concentrate on the molecular markers that are relevant to clinical practice.

Colorectal cancer has been shown to arise through at least two distinct genetic pathways: One involves chromosomal instability (demonstrating high frequency of structural chromosomal changes) and the other involves microsatellite instability (MSI) (demonstrating DNA microsatellites changes).

**Figure 2.**
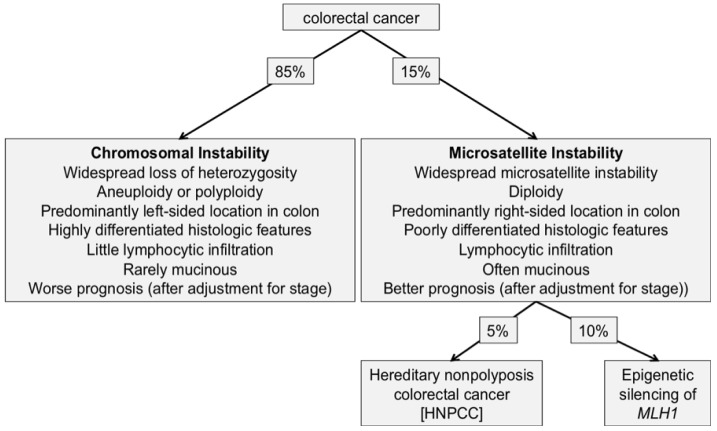
The two genetic pathways of colorectal cancer (modified from de la Chapelle, NEJM 2003 [176]).

The majority of sporadic cases of colorectal cancers (up to 85%) display chromosomal instability (CIN), which manifests as aneuploid and polyploid karyotypes as well as multiple structural chromosomal changes such as translocations, allelic losses, amplifications and mutation of APC, and KRAS, the remainder of colorectal cancer patients (15%) demonstrate MSI [175,176]. This is illustrated in Figure 2.

### 10.2. Microsatellite Instability (MSI)

Microsatellite instability has generated a great deal of interest in the past two decades. Microsatellites are normal segments of DNA with repeat sequences of nucleotides of set length. Change in the number of repeats or its length (as a result of nucleotide insertions or deletions) is called microsatellite instability (MSI which is caused by defective DNA mismatch repair genes (dMMR) [176].

Microsatellite instability in tumors has been classified by the US National Cancer Institute workshop on microsatellite instability for cancer detection in familial predisposition, into three categories, high, intermediate and stable tumors. These are defined as MSI-H (tumors showing instability in 30% or more of the microsatellites), MSI-L (instability in 10%–30%) and MSS (less than 10%) [177].

Patients with MSI-H tumors, have a relatively stable diploid karyotype [178,179]. They typically have right sided or proximal tumors, which, tend to be associated with the following characteristic pathological morphological features: Mucinous adenocarcinoma, poor differentiation, medullary carcinoma, tumor infiltrating lymphocytes TILs (five or more in at least one of 10 HPF), Crohn's-like reaction and serrated adenocarcinoma heterogeneity, but they usually exhibit improved overall survival [180]. It has been reported that some histopathological features has been more frequently encountered in sporadic MSI-H cancers usually lacking MLH1 such as poor differentiation, mucinous subtype and lower stage at presentation as opposed to HNPCC MSI cancers which are more likely to lack MSH2 [181]. Dirty necrosis and tumor budding are more a feature of MSS phenotype and not encountered in MSI-H cancers [74,182]. The most sensitive indicators seem to be TIL and right sided location [183].

As noted earlier, approximately 15% of all colorectal cancers demonstrate MSI, one-third of these cancers are seen in HNPCC and two-thirds are sporadic cancers [184]. Dysfunction of the mismatch repair (MMR) system is either caused by germline mutational inactivation of genes encoding MMR proteins, most commonly MLH1, MSH2, and MSH6 in HNPCC patients or by epigenetic silencing of the promoter region of MMR genes, predominantly MLH1, by CpG island hypermethylation. The latter mechanism is responsible for the vast majority of sporadic colorectal cancers with a high level of MSI [176,185-189].

Testing for microsatellite instability is used for screening for HNPCC as well as assessment of patient's prognosis. MSI status can be assessed by polymerase chain reaction (PCR) using an automated sequencer to amplify specific microsatellite repeats which is compared to the length of repeats obtained from normal DNA extracted from adjacent normal mucosa cells or other normal tissue (blood, buccal smear) [190]. Lack of expression of MMR proteins by immunohistochemistry (IHC) (primarily using antibodies to the MLH1 and MSH2 proteins) is diagnostic for dMMR and is often used in MSI tumor analysis as an alternative to PCR, as well as to complement genetic testing for HNPCC patients [188,191].

A scoring system MsPath has been proposed to assess MSI H status and possible hereditary predisposition (screening for HNPCC) using clinico-pathological features such as age at diagnosis, anatomical site, histological subtype (mucinous, signet ring, undifferentiated), tumor grade, Crohn's like reaction and TILs [183].

MSI status appears to be an important prognostic factor in colorectal cancer. Most, but not all retrospective studies, have shown that colon cancers with MSI-H have better stage-adjusted survival rates compared with MSS tumors, in patients treated with surgery only [180,192,193]. A systematic review of 32 studies including 7642 patients, including 1277 with MSI showed better overall survival in patients with MSI-H tumors. This benefit was maintained when the analysis was restricted to clinical trial patients [194]. Most recent publications continue to show such benefit [195-197].

The relatively excellent prognosis of MSI-H tumors, surprisingly does not seem to be affected by the presence of BRAF V600E mutation (which carry poor prognosis when expressed in MSS) [198].

MSI status may be a predictive marker for lower response rates to 5-fluorouracil chemotherapy. Recent data suggest that patients with MSI-H cancers do not derive survival benefits from treatment by 5-FU based adjuvant chemotherapy unlike their MSS cancers counterparts [193,194,196,197,199]. Other studies do not support this conclusion [192]. As MSI results from loss of critical components, typically the MLH1and MSH2 proteins which are involved in the repair of inter-strand nucleotide mismatches and the loops of DNA that result from a mismatched number of complementary nucleotides. This recognition of distortions in the DNA helix also serves to recognize DNA adducts formed by chemotherapeutic agents which results in resistance to certain chemotherapeutic agents; importantly, resistance to 5-FU [200]. The relative resistance is clinically relevant as it may potentially influence treatment responses based on MSI status. This led some investigators to recommend that stage II colon tumors should be analyzed for MSI status to guide decisions on the use of adjuvant therapy [196,197].

Other investigators have suggested that additional work needs to be done to determine whether adjuvant FU-based chemotherapy regimens are truly a mismatch for stage III patients with high levels of MSI, *i.e.*, whether tumor MSI status can be used as a predictive marker for not using FU-bases adjuvant chemotherapy in these patients [189]. Another consideration relates to the fact that the value of MSI tumor status as a prognostic or predictive marker in the adjuvant setting may be affected by mutations to other genes involved in colon cancer etiology, such the BRAF gene [201].

### 10.3. RAS-RAF-MEK-ERK-MAP Kinase Pathway Mutations

The RAS-RAF-MEK-ERK-MAP kinase pathway is an important cellular signal transduction pathway which mediates cellular responses to growth signals. It thus regulates cell proliferation, cell, survival, cell-cycle arrest, terminal differentiation and apoptosis in response to extracellular signals [202,203]. RAS is a protein that is attached to the inner surface of the plasma membrane, whereas RAF, MEK, and ERK are cytosolic protein kinases that sequentially activate each other, forming a three-tiered signaling cascade [204]. RAS is mutated to an oncogenic form in about 15% of human cancer [202], while B-RAF is mutated (BRAFMUT) in approximately 7% of human cancers [205]. In colorectal cancers the incidence of KRAS or BRAF mutations are about 50% of patients (roughly 40% KRAS and 10% BRAF mutations) and was a found to be associated with poor prognosis in colorectal cancer [206].

Mutations in both KRAS and BRAF stimulate the MEK-ERK-MAP kinase cascade pathway [207]. KRAS signaling is mediated through a protein kinase encoded by BRAF gene which acts as its downstream effector. BRAF and KRAS mutations tend to be mutually exclusive [208,209], the former being reported to be more associated with MSI than MSS tumors with subsequent abrogation of their favorable outcome [198]. The MEK-ERK-MAP kinase pathway is possibly activated differently by the native wild-type B-RAF (BRAFWT), which forms a complex BRAFMUT bind independently of RAS [205].

### 10.4. Kras

Aberrant activation of EGFR pathway, occur by mutational activation of KRAS. The KRAS proto-oncogene encodes guanosine triphosphate/guanosine diphosphate binding protein which transduces signals from activated cell surface receptor to the nucleus, thus point mutation in the KRAS gene in codons 12 and 13 of exon 2 down-regulates GTPase activity resulting in the activation of the RAS-RAF signaling pathway [210]. This initiates a mitogenic signaling cascade, resulting in tumor proliferation, angiogenesis, invasion and metastatic potential [211,212].

EGFR-targeted monoclonal antibodies (cetuximab and panitumumab) have been found to significantly improve disease response and are currently being used in treatment of metastatic colorectal cancer [213]. This occurs only in tumors which have wild type KRAS [214-217].

Thus, testing for KRAS mutations has emerged as an important predictive marker of resistance to anti EGFR agents, panitumumab [217] or cetuximab [215,218]. Testing for KRAS mutations allow oncologists to choose the appropriate patients with metastatic disease for targeted therapy with epidermal growth factor receptor inhibiting (EGFRI) agents such as cetuximab and panitumumab. It is now recommended that KRAS gene testing is done for all patients with metastatic colorectal cancer.

The currently followed practice is to offer treatment with anti EGFR therapy to patients with metastatic colorectal cancer harboring wild type KRAS only [219]. Patients with the mutated KRAS should not receive these therapies.

Analysis of KRAS mutational status is commonly performed on the primary tumor as this mutation occurs early in carcinogenesis, an assumption which was called into question in a study by Oliveria *et al.* in 2007 [220], which found an increased incidence of KRAS mutation in lymph node metastases relative to their primary, hence the need to re-biopsy. However, other studies reported concordance between the primary tumors and their liver and lung metastases [221].

While testing for KRAS mutation is clearly predictive of resistance to anti EGFR agents, its use in prognosis remains unsettled. Some studies reported poorer prognosis including a large multicentre study (3439 patients) which found that glycine to valine mutations involving codon 12 of KRAS was significantly associated with poorer progression free and overall survival in patients with stage III disease, irrespective of treatment [222]. In a recent report, which studied 330 patients with metastatic colorectal cancer, KRAS mutation was also found to be an independent prognostic factor significantly associated with shorter disease free survival but not with overall survival. The latter was attributed to the administration of chemotherapy following relapse [221]. The latter study also demonstrated a higher frequency of KRAS mutation in colonic primary tumors with lung metastases in comparison to those with liver metastases, a finding which was not reproducible in rectal tumors. On the other hand, two recent trials found that there was no association between KRAS mutation and prognosis for patients treated with neo-adjuvant chemotherapy. PETACC-3, an adjuvant trial including patients with stage II to III colon cancer found KRAS mutation in 37% of cases, the incidence of which did not vary with tumor stage but was associated with grade. KRAS mutation did not have a prognostic impact on relapse free survival or overall survival [223]. Similarly, no effect on prognosis was found by CALGB 89803 trial, which studied patients with stage III colon cancer. No predictive value was found to KRAS mutation status and response to standard chemotherapy [224].

### 10.5. Braf

BRAF mutation has been found to be an independent prognostic factor for decreased survival in colorectal cancer patients on univariate as well as multivariate analysis [206,216,224,225]. As mentioned earlier, this poor prognostic effect occur in MSI-L and MSS tumors [198,223] It is also associated with resistance to anti-EGFR monoclonal antibodies (cetuximab and panitumumab) [214,216].

Souglakos and associates [216] demonstrated that BRAF mutations in primary colorectal cancer mark patients with poor prognosis regardless of specific treatment regimen. Patients with BRAF mutation had significantly higher likelihood of disease progression (P < 0.0001) or death (P < 0.0001) with any treatment regimen. The BRAF V600E mutation predicted independently early relapse on first-line therapy and death. It was deduced that BRAF mutation does not simply substitute for KRAS activation in a linear signaling pathway but probably confers distinct impact on prognosis. It also suggests that KRAS mutation may bypass aberrant EGFR signaling.

In the PETACC-3 study which included stage II and stage III cancers, BRAF tumor mutation was found in 7.9% of cases and there was no significant variability with tumor stage. In a multivariate analysis, BRAF mutation was significantly associated with female sex, and highly significantly associated with right-sided tumors, older age, high grade, and MSI-high tumors. In univariate and multivariate analysis BRAF mutation was not prognostic for relapse free survival but was prognostic for overall survival, particularly in patients with MSI-L MSS tumors [223].

As a predictive marker, patients with BRAF mutant tumors treated with cetuximab had also lower progression free survival compared with those with BRAF wild type (0 *vs.* 17%) in the study by Souglakos *et al.* in 2009 [216], a finding which could partly explain resistance to anti EGFR targeted therapy in a subset of patients with tumors harboring KRAS wild type. This is in keeping with an earlier study by Di Nicolantonio and colleagues [214], where the response to panitumumab or cetuximab was found to be impeded by the presence of BRAF V600E mutation and restored (in a cellular model of CRC cells) by BRAF inhibitor sorafenib [214]. They suggested that this experimental observation should encourage conceiving clinical trials using multiple therapies with EGFR and BRAF/MAPK inhibitors, considering that cetuximab, panitumumab, and sorafenib are already approved for clinical use.

Standard neoadjuvant chemotherapy, using irinotecan or oxaliplatin did not seem to be affected by KRAS/BRAF mutations [206].

### 10.6. PI3K and PTEN

PI3KCA mutation and PTEN deletion are two promising biomarkers that may predict resistance to anti EGFR therapy (e.g., cetuximab or panitumumab). Phosphatidylinositol 3-kinase (PI3K) is another pathway for down-stream signaling and activation stimulated by EGFR in addition to Ras/Raf/MAPK. Molecular alterations in that pathway, which in colorectal cancer are mainly mutations in PIK3CA and loss of PTEN protein expression, have been proposed as additional markers for anti-EGFR therapy resistance. The significance of alterations in this pathway in relation to resistance to anti EGFR therapy is not yet established; nevertheless the evidence suggests a possible negative predictive role for EGFR monoclonal antibody-based treatment [226]. Both PIK3CA mutations and PTEN loss may co-exist with KRAS or BRAF mutations. When PIK3CA mutations and PTEN loss of expression are combined with KRAS and BRAF mutations, up to 70% of patients may be non-responsive to cetuximab or panitumumab. It has been suggested that colon cancer may be typed and termed ‘quadruple-negative’ for patients who do not have mutations in any of these four markers [227].

### 10.7. CPG Island Methylator Phenotype

Aberrant hypermethylation of DNA is common in human cancers and has been associated with silencing of important tumor-suppressor genes [228], a phenomenon which has been described in colorectal cancer [229]. Those with high degrees of methylation (CIMP) were found to represent a distinct subtype with specific precursor lesions, clinical characteristics, molecular associations and morphological features. They evolve through a different pathway with sessile serrated adenomas as their precursor lesion, while cancers with low methylation are said to arise from traditional serrated adenomas or classic adenomas [102]. CIMP cancers seem to be more common in proximal tumors, in women, and in older patients [230]. Histologically, they show serration, sheeting, mucin production poor differentiation, Crohn's like reaction, tumor-infiltrating lymphocytes and lack necrosis. CIMP tumors that lack methylation of MLH1 are microsatellite stable, poorly differentiated with discohesive pleomorphic cells invading the stroma, exhibit bizarre nuclei, cribriform architecture with central necrosis and lack serrated pattern as well as tumor infiltrating lymphocytes. It shows high rate of microsatellite instability and BRAF gene mutation is common, while p53 is low [230-232].

The literature lacks consistency regarding the prognostic and predictive significance of CIMP colorectal cancers, which was partly explained by the lack of consensus regarding the methylation markers, used to define CIMP as well as the heterogeneity within CIMP, those with hypermethylation of MLH1 and those who lack it and type of chemotherapy given.

In one study, CIMP in stage III colorectal cancers it was found that methylation predicted better prognosis [233], however, the CIMP cases in that study were largely from patients with hypermethylation of hMLH1 and MSI. In a recent study by Ogino *et al.* in 2009 [234], which included stages I-IV cancers, CIMP-high tumors were associated with a significant reduction in colon cancer-specific mortality, regardless of both MSI and BRAF status. The relation between CIMP-high and lower mortality appeared to be consistent across all stages.

On the other hand, it was reported that within the microsatellite stable MSS group hypermethylation was associated with proximal location, BRAF mutation and shorter survival [235]. In an earlier report by Shen *et al.* in 2007 [236] following patients with advanced colorectal cancer treated with 5-fluorouracil based chemotherapy, concurrent methylation of two or more genes of the CIMP defined a group of cases with markedly reduced overall survival in multivariate analyses.

### 10.8. Thymidylate Synthase

The thymidylate synthase gene encodes enzyme thymidylate synthase (TS) that catalyzes pyrimidine synthesis an essential component of DNA synthesis. It is also a target for 5-FU (pyrimidine analogue) resulting in cell death and apoptosis. High expression of thymidylate synthase gene has been associated with tumor recurrences in stage II and stage III colon cancer [237]. Polymorphisms in the thymidylate synthase gene (TYMS) defines two risk groups associated with dissimilar tumor disease survival rates (60% *vs.* 22%) [238].

Tan and associates [239] selected the type of adjuvant therapy prospectively in 135 T3/T4 rectal cancer patients, who were stratified according to their germline TYMS genotyping. Patients with low-expression genotypes (98 patients) were considered good risk and were treated with chemoradiotherapy using infusional FU, while those with high expression genotypes (37 patients) were considered poor risk and treated with FU/RT plus weekly intravenous irinotecan. Results showed that high rates of disease survival and pathological response (tumor down staging), were achieved among both risk groups when personalized treatment was based on TYMS genotype, with DS and ypT0 rates reaching 64.4% and 20% for good-risk and 64.5% and 42% for poor-risk patients, respectively. The clinical value of TYMS gene expression testing remains to be verified by further larger studies.

### 10.9. 18qLOH

Other promising biomarkers with potential prognostic significance include; allelic imbalance on chromosome 18q [219]. Loss of heterozygosity (LOH) at chromosome 18q frequently occurs late in colorectal cancer and is inversely associated with microsatellite instability [240]. It is unclear whether 18q deletions or loss of heterozygosity (18qLOH) represents an independent prognostic marker, or is merely a surrogate marker for CIN/MSS colorectal cancers [241]. Patients with colorectal cancer with 18qLOH are generally associated with poor prognosis, compared with patients with tumors that do not carry 18qLOH [242]. A meta-analysis of 17 independent studies, found the overall hazard ratio (HR) of poor prognosis to be 2 for patients with 18qLOH and HR of 1.69 in the adjuvant setting [243]. The independent prognostic contribution of 18q deletion or 18qLOH in colorectal cancer has been called into question by a recent prospective study on 555 non-MSI-high colorectal cancers, that found no difference in prognosis attributable to 18qLOH [240]. Loss of 18q has been associated with poor response to 5-FU-based adjuvant chemotherapy [242].

### 10.10. Tumor Tissue CEA

High Tissue CEA concentration may be a useful and independent predictor for poor outcome [244].

### 10.11. Cancer Stem Cell Markers and Prognosis

An interesting new concept suggests that cancer progression and resistance to chemotherapy may be related to the presence of a small subset of cells within solid tumors which have “stem cell-like” characteristics. These cancer stem cells have high self-renewal capacity, ability to differentiate into active proliferating tumor cells, and resistance to apoptosis and chemotherapy or radiation [244].

### 10.12. Summary of Commonly Used Prognostic and Predictive Molecular Markers

There are many prognostic and predictive molecular biomarkers in colorectal cancer. Some are now in clinical use, while others show promise for future use. The most commonly used biomarkers are summarized in Table 3.

Prognostic markers identify patients at higher risk of recurrence, independent of treatment and are useful in selecting patients who should receive adjuvant chemotherapy. Predictive markers, on the other hand identify patients likely to benefit from a specific type of chemotherapy, *i.e.*, useful in selection of drugs most likely to be effective.

## 11. Conclusions

This review of prognostic features of colorectal carcinoma is by no means exhaustive, but sheds light on the established and most recently recognized areas in which the pathologist contributes to the prognostic stratification of these patients to help guide their management. The emergence of novel methods to improve prediction of clinical behavior has contributed significantly to this exciting and rapidly developing field. Molecular testing is opening a number of new therapeutic avenues, and this needs to be incorporated into information gleaned by the traditional, still valuable, histological analysis. Further study in this area will continue to refine prognostic information and help govern new therapeutic interventions.

## Figures and Tables

**Table 1.** Count of medical subject headings used in literature search.

**Medical Subject Headings**	**Articles**	**Reviews**
Colorectal cancer and Prognosis	16056	2551
Colorectal cancer and Prognostic	4817	596
Colorectal cancer and Prognostic factors	2223	256

**Table 2.** Comparison of the last three editions of TNM Classification.

**TNM Edition**	**5th (1997)**	**6th (2002)**	**7th (2009)**
T		**T4a** directly invades other organ or structures **T4b** invade visceral peritoneum	**T4a** perforates visceral peritoneum **T4b** directly invades other organ or structures
N	**N1**: 1–3 positive nodes **N2**: 4 or more positive nodes	N1: 1–3 positive nodes N2: 4 or more positive nodes	N1: 1–3 positive nodes **N1a**: 1 node **N1b**: 2–3 nodes **N1c**: satellites in subserosa, without regional nodes * N2: 4 or more positive nodes **N2a**: 4–6 nodes **N2b**: 7 or more nodes
Isolated tumor cells (ITCs) §		ITC considered as N0˜	ITC considered as N0˜
Nodal Micrometastasis *		Considered as N1˜	Considered as N1˜
Tumor Deposits (TD)	introducing the 3-mm rule	replacing the 3-mm rule with the contour rule	
M			**Mx** removed **M1a** one organ **M1b** >one organ or peritoneum
Stage Grouping		**Stage II** is subdivided into IIA and IIB based on whether the primary tumor is T3 or T4,respectively Stage III is subdivided into IIIA (T1-2N1 M0), IIIB (T3-4 N1 M0) or IIIC (any T N2 M0)	**Stage II** is subdivided into IIA, IIB andIIC based on whether the primary tumor is T3, T4a or T4b, respectively **Stage III** is subdivided into IIIA, IIIB and IIIC IIIA limited to T1, T2, N1, N2a M0 III B any N2b M0 any T except T4b IIIC any T4b N1 N2 M0 **Stage IV** is subdivided into IVA, and IVB based on whether metastases are M1a or M1b respectively

Box with yellow shading: 6th Edition supplement [23];

§ : ITC defined as small numbers of tumor cells detected only by special techniques or seen histologically, but measuring <0.2 mm;

* : Micrometastasis defined as metastatic tumor that measure >0.2 mm, but <2.0 mm;

˜ : The number of lymph nodes involved by micrometastasis or ITCs should be clearly stated.

**Table 3.** Prognostic and Predictive molecular markers in colorectal cancer.

**Molecular Marker**	**Prognostic value**	**Predictive value**	**Comment**
KRAS	Likely * unfavorable in advanced disease [221,222]	Predicts resistance to anti-EGFR therapy (cetuximab and panitumumab) [215,217,218]	Found in up to 40% of CRC [206]. Now used to predict response to cetuximab and panitumumab [219].
BRAF V600E mutation	Likely * unfavorable [206,216,224,225]	Appear to predict resistance to anti-EGFR Therapy [216]. May predict response to BRAF inhibitors [214]	Found in up to 10% of CRC [206].
PIK3CA mutations	Likely unfavorable [227]	Appear to predict resistance to anti-EGFR therapy [226,227]	Promising
PTEN loss	Likely unfavorable [227]	Appear to predict resistance to anti-EGFR therapy [226,227]	Promising
Microsatellite instability (MSI)	Favorable prognosis and overall survival in patients with MSI-H tumors [180,192,193,198]	Patients with MSI-H cancers do not derive survival benefits from treatment by 5-FU based adjuvant chemotherapy [193,194,196,197,199]	Found in up to 15% of CRC [175,176]. Not yet in routine clinical use as a predictive biomarker
18q deletions or loss of heterozygosity (LOH)	Unfavorable prognosis, but may not be independent of CIN/MSS [241]	May predict resistance to 5-FU [242]	Clinical value remains to be determined, as evidence not yet conclusive
Thymidylate synthase (TS) expression	High thymidylate synthase expression may be associated with tumor recurrence in stage II and III colon cancer [237] ^§^	Low thymidylate synthase (TS) levels are associated with better clinical response to fluorouracil-based chemotherapy and higher risk of toxicity [238,239]	Clinical value remains to be determined, as evidence not yet conclusive

* Some inconsistent evidence;

§ Some conflicting evidence.
